# Interaction between Uranyl Cations and Layered Double Hydroxide Nanoparticles: Implications for Nuclear Wastewater Management

**DOI:** 10.1021/acsestwater.4c00313

**Published:** 2024-06-11

**Authors:** Tamás Péter, Dóra Takács, Szilárd Sáringer, Adél Szerlauth, Kadosa Sajdik, Gábor Galbács, Matija Tomšič, Samuel Shaw, Katherine Morris, Grant Douglas, István Szilágyi

**Affiliations:** † MTA-SZTE Lendület Biocolloids Research Group, Interdisciplinary Excellence Centre, University of Szeged, H-6720 Szeged, Hungary; ‡ Department of Physical Chemistry and Materials Science, University of Szeged, H-6720 Szeged, Hungary; § Department of Molecular and Analytical Chemistry, University of Szeged, H-6720 Szeged, Hungary; ∥ Faculty of Chemistry and Chemical Technology, University of Ljubljana, Večna pot 113, SI-1000 Ljubljana, Slovenia; ⊥ Research Centre for Radwaste Disposal and Williamson Research Centre, Department of Earth and Environmental Sciences, University of Manchester, U.K.-M139PL Manchester, United Kingdom; # Centre for Environment and Life Sciences, CSIRO Environment, WA-6913 Wembley, Australia; ∇ School of Molecular and Life Sciences, Curtin University, WA-6102 Bentley, Australia

**Keywords:** layered double hydroxide, uranyl, precipitation, aggregation, remediation, colloidal stability

## Abstract

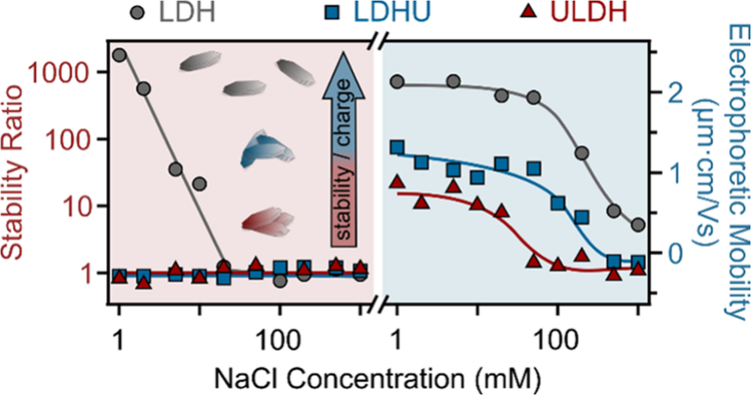

Effective uranium (U) capture is required for the remediation of contaminated solutes associated with the nuclear fuel cycle, including fuel reprocessing effluents, decommissioning, or nuclear accident cleanup. Here, interactions between uranyl cations (UO_2_^2+^) and a Mg–Al layered double hydroxide (LDH) were investigated using two types of uranyl-bearing LDH colloids. The first (ULDH) was synthesized by coprecipitation with 10% of Mg^2+^ substituted by UO_2_^2+^. Alternatively, UO_2_^2+^ was added to a neoformed LDH to obtain the second uranyl-bearing LDH colloid (LDHU). In both the LDHU and ULDH colloid systems, schoepite (UO_2_)_8_O_2_(OH)_12_·12H_2_O, was formed. The presence of U significantly reduced the size of both LDHU and ULDH compared to a reference LDH colloid. Surface charge and aggregation of the ULDH and LDHU colloids were compared in NaCl, Na_2_CO_3_, Na_2_SiO_3_, and Na_3_PO_4_ solutions that are often present in nuclear wastewaters. Aggregation of ULDH and LDHU in the presence of Na_2_SiO_3_ or Na_3_PO_4_ promotes colloid restabilization. While the uranyl cation was not incorporated into the LDH structure, it influences nanoparticle growth in addition to imparting modified surface properties that affect aggregation. This has implications for radioactive waste disposals, where LDH, which can also incorporate a variety of other radionuclides, is used for remediation.

## Abstract 2

Results from various independent techniques revealed that layered double hydroxides are efficient adsorbents for remediation of uranium-contaminated waters.

## Introduction

Uranium (U) present in wastewaters produced via its mining, processing, and use in the nuclear fuel cycle is a significant environmental concern^1,2^ with a range of potential ecotoxicological effects identified.^3,4^ This includes the presence of U in the remediation of contaminated solutes produced during various processes associated with the nuclear fuel cycle, including U-mining, effluents from spent nuclear fuel reprocessing, nuclear fuel storage facilities,^5,6^ U enrichment sites that are subject to decommissioning,^7,8^ or cleanup procedures following the nuclear accidents.^9,10^ To address these challenges, and especially when other radionuclides including transuranics such as plutonium and americium isotopes, and fission products including ^90^Sr and ^137^Cs may also be present, several studies have investigated U capture^11^ using various materials^12−19^ including layered double hydroxides (LDHs) formed in situ^20−23^ or prefabricated LDHs as adsorbents.^17,24−27^

The utility of LDH minerals, e.g., Mg–Al-rich hydrotalcite group LDH ([Mg_1–*x*_Al_*x*_](OH)_2_A_*x*/m_^m–^·*n*H_2_O, A = anion), is the prospect of simultaneous capture of U uranyl oxycation (UO_2_^2+^) and other radionuclides whether present as (oxy)cations or (oxy)anions.^28^ Previous studies, however, have indicated that the UO_2_^2+^ oxycation is sterically hindered, particularly where common ligands (e.g., carbonate and sulfate) may also be present, preventing its incorporation within both the metal hydroxide layers and interlayers (as anionic complexes e.g., UO_2_(CO_3_)_2_^2–^) of LDH. Rather, it is adsorbed at the layer periphery (e.g., adsorbed to edge sites) where its binding may be facilitated by interaction with carbonate ligands.^23^ Little is currently known about how the presence of elevated concentrations of UO_2_^2+^ may influence the fundamental particle and colloidal properties of LDHs, including particular size, morphology, crystallinity, surface charge/electrophoretic mobility, and aggregation behavior in the presence of common anions known to interact with LDH via either interlayer anion exchange or surface/edge bonding.

In the present study, building on similar previous investigations of U-colloid interaction,^29,30^ we investigate the interaction of UO_2_^2+^ (0.01 M as UO_2_(CH_3_CO_2_)_2_) with Mg–Al LDH colloids under two different conditions; first, where LDH was formed via coprecipitation in the presence of 10% of the stoichiometrically required Mg^2+^ substituted by UO_2_^2+^ (ULDH), and the second where the UO_2_^2+^ was added to a neoformed LDH precipitate (LDHU). The colloid chemistry and behavior of the two uranyl-bearing LDH systems were then compared with a similarly prepared reference UO_2_^2+^-free LDH.

## Methods

### Materials

Analytical-grade inorganic compounds (Mg(NO_3_)_2_·6H_2_O, Al(NO_3_)_3_·9H_2_O, NaOH, UO_2_(CH_3_CO_2_)_2_, NaCl, Na_2_CO_3_, Na_3_PO_4_, and Na_2_SiO_3_) were procured from VWR and used without further purification. Ultrapure water (Adrona) was used for the preparation of all LDH samples.

### Preparation of Layered Double Hydroxides (LDHs)

Three distinct types of LDH were prepared for this study. The first, an unmodified Mg–Al LDH served as a reference material where no UO_2_^2+^ was present. This was prepared by dissolving the metallic salts in water (0.3 M Mg(NO_3_)_2_ and 0.1 M Al(NO_3_)_3_), and the pH was set to 10 with a 4.0 M NaOH solution. The resulting LDH suspension was vigorously stirred for 30 min in an N_2_ atmosphere. Thereafter, the sample was centrifugated at 4200 rpm for 15 min, resuspended, and washed multiple times with water to remove residual dissolved salts.

The second type of LDH was synthesized via coprecipitation, where 10% of the stoichiometric requirement of Mg^2+^ was substituted by UO_2_^2+^ (ULDH). Accordingly, 0.29 M Mg(NO_3_)_2_, 0.1 M Al(NO_3_)_3_, and 0.01 M UO_2_(CH_3_CO_2_)_2_ were dissolved in 50 mL of water, and the pH was set to 10 with a 4.0 M NaOH solution. The remaining steps of the process aligned with the reference LDH synthesis.

For the third sample (LDHU), following the preparation of LDH, an aliquot of 0.01 M UO_2_(CH_3_CO_2_)_2_ solution was introduced to 50 mL of a pH 10 neoformed LDH suspension, resulting in a Mg/Al/U molar ratio of 2.9:1.0:0.1. The suspension was then gently stirred for an additional 30 min. Subsequently, the sample was centrifuged at 4200 rpm for 15 min and then washed multiple times with water to remove residual dissolved salts.

After synthesis, the Al, Mg, and U content in the dried LDH, ULDH, and LDHU solids was determined by dissolving the samples in 10% HCl, and the solutions were analyzed by an inductively coupled plasma mass spectrometer (ICP-MS, Agilent 7700X). The theoretical and experimentally obtained metal ion contents of the LDH, ULDH, and LDHU are shown in Table S1 in the Supporting Information (SI).

### Characterization Techniques

The LDH, ULDH and LDHU mineralogy was analyzed by X-ray diffraction (XRD) using a Bruker D8 Advanced diffractometer with Cu Kα radiation source with a wavelength (λ) of 0.1542 nm. The diffraction beam was detected over a scattering angle 2θ range of 5–80° with a step size of 0.02°.

The nanoscale structure of the studied powders was also investigated using both small-angle X-ray scattering (SAXS) and small- and wide-angle X-ray scattering (SWAXS), performed with a laboratory-modified old-Kratky type camera (Anton Paar) with focusing multilayer optics and a block-collimation unit to obtain a well-defined focused Cu–Kα line. The SAXS scattering curves were acquired with a Mythen 1K microstrip solid-state diode-array detector (Dectris) in the range of scattering vector (*q*) from 0.098 to 7.0 nm^–1^ (0.00657 nm^–1^ step), where *q* = (4π/λ)sin(θ),^31^ and were corrected for sample X-ray absorption and background scattering (sample holder) and transformed to absolute scale using water as a secondary standard.^32^ The SWAXS 2D images were acquired by the 2D-imaging plate system Fuji BAS 1800II with a spatial resolution of 50 × 50 μm^2^ per pixel.

Further information on the LDH, ULDH, and LDHU structures was obtained from Raman spectra, which were recorded with a Bruker Senterra II Raman microscope at an excitation wavelength of 785 nm applying 100 mW laser power and averaging 128 spectra with an exposition time of 16 s.

The morphology of the LDH, ULDH, and LDHU was characterized by Transmission Electron Microscopy (TEM) and Atomic Force Microscopy (AFM). TEM images were obtained using an FEI Tecnai G2-type electron microscope. LDH, ULDH, and LDHU nanoparticle dispersions were dried on a Cu–C mesh grid, and 200 kV accelerating voltage was used for imaging in bright-field mode. AFM images were collected with a Multimode Nanoscope IIIa AFM instrument. The device was used in tapping mode in the air at ambient temperature using a Si tip cantilever (Veeco Nanoprobe Tips RTESPA model). The dispersions were deposited on a freshly cleaved mica substrate (Ted Pella, Highest grade V1) prior to imaging.

Dynamic light scattering (DLS) was used to measure the hydrodynamic radius of the dispersed nanoparticles.^33,34^ The measurements were carried out with a compact goniometer system (ALV/CGS-3) at a 90° scattering angle and borosilicate cuvettes (Kimble Chase). The correlation function was accumulated for 20 s, and the cumulant method was used to obtain the decay rate and, subsequently, to determine the hydrodynamic radius.^35^ During sample preparation, a calculated amount of salt solutions, water, and particle dispersions were mixed to achieve the desired salt and particle concentrations. The final volume used was 2 mL, with the particle dose set to 10 mg/L.

The electrophoretic mobility (EM) of LDH, ULDH and LDHU colloids was measured with a Litesizer 500 (Anton Paar) device equipped with a 40 mW laser source operating at a 658 nm wavelength. The sample preparation was the same as in the DLS measurements with the exception that the samples were allowed to equilibrate for 2 h at room temperature before measurement, which occurred after 1 min equilibration time in capillary cuvettes.

## Results and Discussion

### LDH Composition

Quantitative analysis of the three LDH types indicates final Mg/Al ratios of 3.1:1.0, 3.0:1.0 and 2.9:1.0 for the LDH, ULDH, and LDHU, respectively, which are in good accordance with the theoretical ratios (Table S1). Importantly, U was quantitatively associated with the ULDH and LDHU samples although may not be necessarily incorporated into the LDH phase, as discussed later.

### Structural Characterization in Solid State

**Figure 1:**
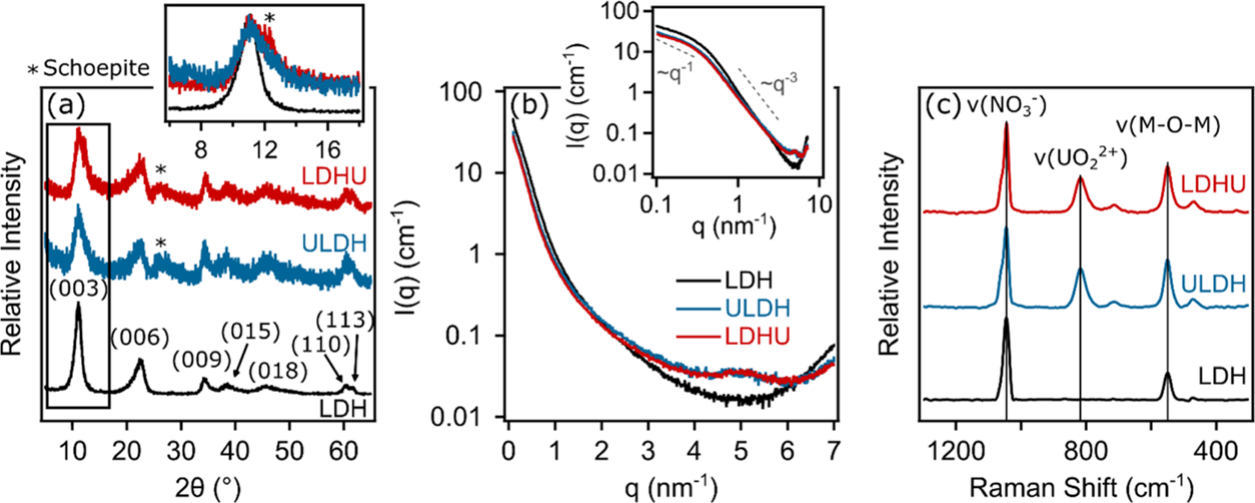
(a) XRD diffractograms, (b) SAXS curves on an absolute scale, and (c) Raman spectra of the LDH, ULDH, and LDHU samples. The inset in (a) shows the (003) reflections in the 6–18° 2θ range, and in (b) the experimental SAXS curves for LDH, ULDH, and LDHU samples in a double logarithmic scale representation.

The three LDH types were investigated by X-ray diffraction (XRD) and Raman spectroscopy to investigate their structure, mineralogy, and composition. The XRD patterns are shown in Figure 1a.

While it was confirmed that LDH formed during all three synthesis methods, for ULDH and LDHU samples, additional reflections appeared around 12 and 26° 2θ indicating the presence of a new phase (labeled with * in Figure 1a) corresponding to schoepite [(UO_2_)_8_O_2_(OH)_12_·12H_2_O].^36,37^ The presence of schoepite indicates no or incomplete incorporation of the UO_2_^2+^ into the metal hydroxide layers either during coprecipitation (ULDH) or via surface cation exchange (LDHU).

To further investigate differences in mineralogy, SAXS was used. The SAXS method has already proved useful in studies of similar colloidal U systems.^38,39^ The data in Figure 1b on a semilog plot (and the inset on a log–log plot) covers the *q* range from 0.098 to 7 nm^–1^, corresponding to the scattering angle 2θ from 0.14 to 9.86°. At these low *q* ranges, information about the nanoscale structural features corresponds to spatial correlations at distances from about 64 to 0.9 nm.

Two interesting features can be seen from these SAXS curves. First, the LDH sample data show a steeper increase in scattering intensity at lower *q* values than the ULDH and LDHU samples and have higher absolute values of the scattering intensity. Since U has a very high electron density compared to Mg and Al as the other major structural elements, one would expect the ULDH and LDHU colloids of comparable size to have higher scattering contrast than the reference LDH. Thus, the higher absolute scattering intensity indicates that the LDH nanoparticles must effectively be larger than the corresponding ULDH and LDHU nanoparticles. Moreover, these steep intensity increases at low *q* values also indicate that the effective total size of the scattering particles in the LDH samples is outside the experimental resolution of our SAXS data. Nevertheless, they obviously show some nanoscale structural details that are still within the resolution of our experiment.

The second feature of interest in the SAXS curves (Figure 1b) is the additional scattering peak at 4.88 nm^–1^, corresponding to a 2θ-value of 6.86° and the real space correlation distance of about 1.3 nm, which is present in the ULDH and LDHU, but not in the LDH scattering curve. The presence of this scattering peak could already be inferred from the XRD spectrum data around the lower 2θ angles, but it is only clearly seen in the SAXS curves. It reveals an additional structural feature with an effective correlation length of 1.3 nm present in the ULDH and LDHU samples. Besides the phase * in Figure 1a, these additional scattering peaks can only be associated with the structural influence or the presence of UO_2_^2+^ in the ULDH and LDHU samples.

In addition, SAXS curves from Figure 1b are shown in a double logarithmic scale in experimental (see Figure S1a in the online SI and the inset in Figure 1b) and desmeared (see Figure S1b in the online SI) forms.^40,41^ The slopes of these SAXS curves indicate the presence of large flat scattering particles (outside the experimental resolution of presented SAXS data) in all three samples studied (LDH, ULDH, and LDHU).^40^ This corresponds to the large original LDH particles and smaller aggregates in the LDHU and ULDH samples. Similarly, SWAXS 2D data (Figure S2) indicate that these powder samples are optically isotropic in that the crystallites are small and are spatially well averaged over all directions.

Raman spectroscopy (Figure 1c) also confirms the presence of UO_2_^2+^ cations associated with the ULDH and LDHU with a peak at 820 cm^–1^, consistent with the U–O stretching vibration of the UO_2_^2+^ bond.^36,42^ Vibration bands were consistent with the presence of NO_3_^–^ as the interlayer ion, as per the metal NO_3_^–^ salts used during synthesis. Besides, the M–O–M vibrations within the metal hydroxide layers are also present in the LDH, ULDH, and LDHU samples. Peak assignments are listed in Table S2.

**Figure 2:**
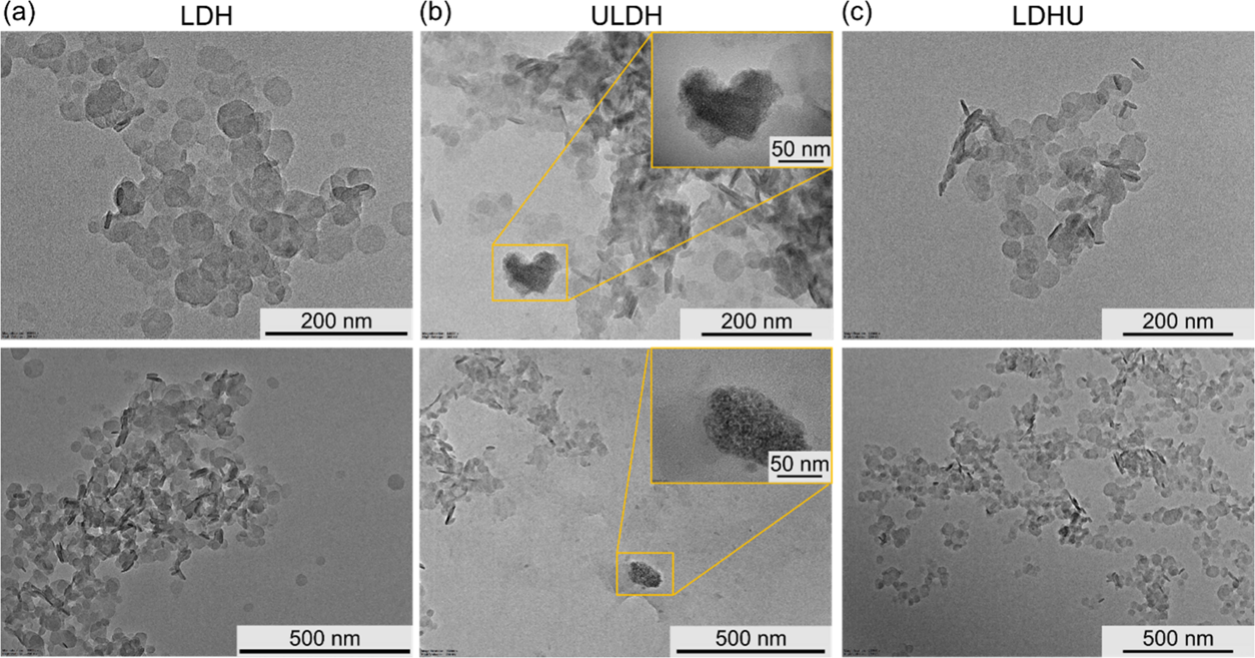
TEM images of (a) LDH, (b) ULDH, and (c) LDHU recorded in a dried stage.

A similar morphology of the LDH, ULDH, and LDHU precipitates is apparent in TEM images (Figure 2), with the precipitates present as aggregates of smaller nanoparticles. The LDH in particular displays characteristic flat, hexagonal platelet-like structures with maximum dimensions of around 50 nm (Figure 2a). Both ULDH (Figure 2b) and LDHU (Figure 2c) have a maximum dimension of about 30 nm. This TEM analysis is consistent with SAXS data, indicating the presence of platelet-like particles in all samples (see Figure S1). Within the ULDH TEM, however, complex worm-like crystal aggregates are also present, which are often observed in LDH precipitates.^22,43^ There is also the possibility that these aggregates in ULDH and LDHU samples may include schoepite, perhaps as a nucleation/aggregation site, as identified in the XRD analysis.

**Figure 3:**
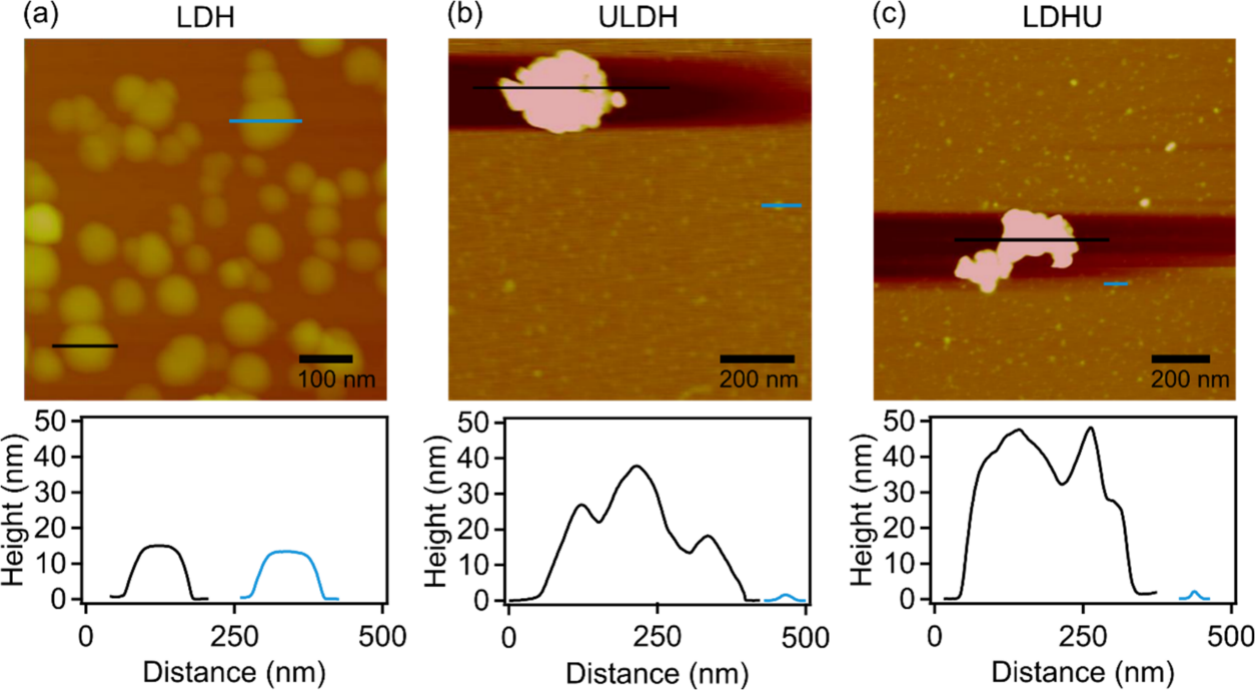
AFM amplitude images and the height profiles (taken along the indicated lines) for (a) LDH, (b) ULDH, and (c) LDHU samples.

Atomic force microscopy (AFM) imaging results confirm the presence of nanoparticles in the LDH sample (Figure 3a) and also the presence of much smaller nanoparticles together with the large nanoparticle aggregates in ULDH (Figure 3b) and LDHU (Figure 3c) samples, as observed in TEM analysis. Similarly, this shows that the size of nanoparticles in LDH prepared in the presence of UO_2_^2+^ cations is much smaller (see Figure 3b,3c blue lines) than in the reference LDH sample (see Figure 3a blue line), even though the large aggregates in ULDH and LDHU materials seem to be much bigger and thicker than the nanoparticles in the LDH sample (see black lines in Figure 3a). These results agree with the results from TEM that the crystals in the ULDH and LDHU samples are smaller than those in the LDH and are in agreement with the SAXS data. The height profile AFM results show that the thickness of the small nanoplatelets of ULDH and LDHU appears to be between 1 and 2 nm, which is consistent with the occurrence of the scattering peak at 4.88 nm^–1^ in SAXS corresponding to a structural feature with a correlation length of 1.29 nm.

Accordingly, it is evident from these findings that the presence of the UO_2_^2+^ cations has an inhibitory effect on crystal growth^23^ during ULDH synthesis, but that it may also correspondingly inhibit further growth of the LDH where UO_2_^2+^ cations are added after formation as in the case of LDHU. In addition, while the shape and thickness of the reference LDH were relatively uniform, those formed in the presence of UO_2_^2+^ cations resemble multiple face-to-face and edge-to-face LDH aggregates, typically 3–4 times thicker, reflecting the likely irregular stacking.

### Colloidal Behavior

**Figure 4:**
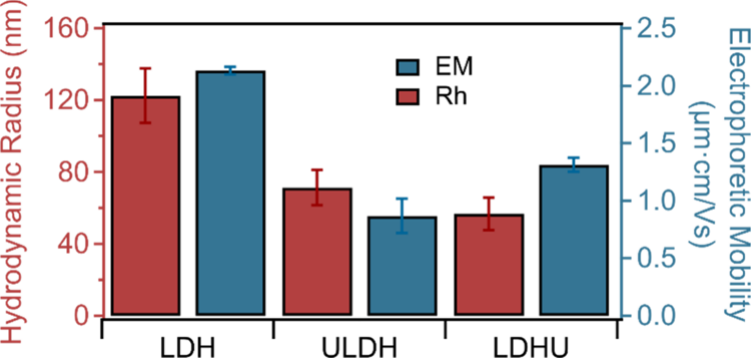
Hydrodynamic radius and electrophoretic mobility data of LDH, ULDH, and LDHU nanoparticles in 1 mM NaCl solutions.

Electrophoretic^44^ and dynamic light scattering (DLS)^33^ techniques were used to investigate the charging and aggregation properties of the LDH, ULDH, and LDHU nanoparticles. The hydrodynamic radius (*R*_h_) and electrophoretic mobility in the presence of 1 mM NaCl for all three types of LDH nanoparticles are shown in Figure 4.

The trend in nanoparticle sizes is consistent with that acquired by TEM and AFM. The largest *R*_h_ of 120 nm, which includes water molecules associated/entrained with the nanoparticles, was for the LDH precipitate, relative to the ULDH and LDHU precipitate of 70 and 50 nm, respectively.

The electrophoretic mobilities of all LDH precipitates were positive, i.e., 2.1, 0.9, and 1.3 μm·cm/(V s) for the LDH, ULDH, and LDHU materials, respectively. The lower mobilities for ULDH and LDHU may reflect the irregular stacking of the nanoparticles such that there was a partial loss of exposed edge charge or the presence of schoepite, which should be negatively charged under the experimental pH studied due to the deprotonation of surface hydroxyl groups.^45,46^

**Figure 5:**
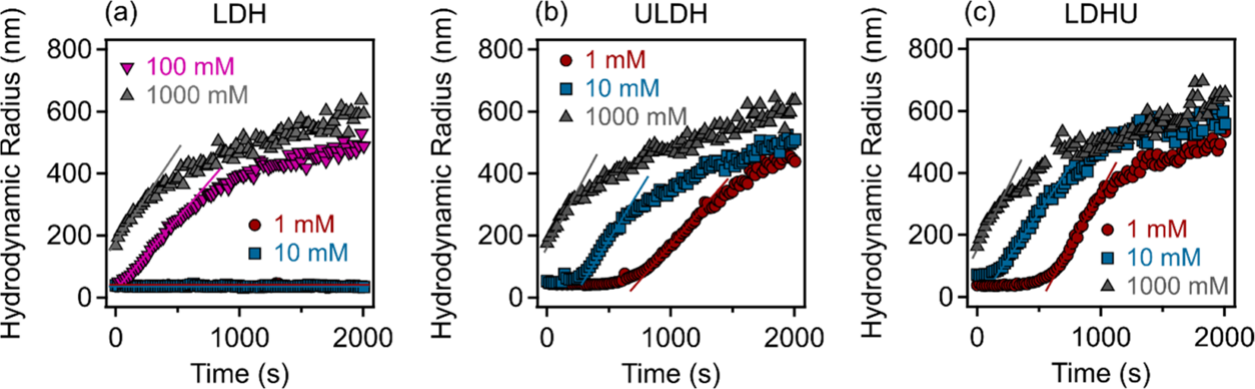
Time-resolved hydrodynamic radii at different NaCl concentrations measured by DLS for (a) LDH, (b) ULDH, and (c) LDHU. The nanoparticle concentration (10 mg/L) was kept constant in the experiments. The solid lines are linear fits used for the stability ratio calculations.

Many U-bearing wastewater may be highly saline, e.g., nuclear fuel reprocessing effluents.^29^ To understand the mechanism of salt-induced aggregation of the three LDH types, time-resolved DLS^33,47,48^ measurements were carried out, in which the LDH nanoparticle suspension concentration was kept constant (10 mg/L), while that of the NaCl electrolyte was systematically varied. The results indicate that the time evolution of the average *R*_h_ is strongly dependent on the electrolyte concentration (Figure 5).

In the case of the reference LDH-NaCl system (see Figure 5a), at lower ionic strengths (1 and 10 mM), the dispersion was stable, with the average *R*_h_ consistent over time. After increasing the electrolyte concentration beyond a system-dependent threshold NaCl concentration (100 mM), the time evolution of *R*_h_ can be divided into two distinct domains. No change could be observed within an initiation period of a few tens of seconds, which is progressively reduced as the electrolyte concentration is increased to 100 mM and then 1000 mM. Thereafter, the *R*_h_ increases sharply followed by a similar reduced rate of increase corresponding to the increasing NaCl concentration after ca. 600 and 400 s, respectively. An asymptote to a stable radius is not attained even after 2000 s for these two highest NaCl concentrations in the reference LDH systems.

In the case of the ULDH, for both 1 and 10 mM NaCl concentrations, there is a time lag of system stability of ca. 600 and 300 s, respectively, before an increase in *R*_h_ occurs (see Figure 5b). Thereafter, there are sharp increases in *R*_h_ elevating in rate with corresponding increasing NaCl concentrations, which slow at ca. 1200 and 600 s, respectively. As for the LDH, the ULDH does not display a time lag for an increase in *R*_h_ in the 1000 mM NaCl system. Thus, at 1 and 10 mM NaCl concentrations, the ULDH defines three distinct domains of initiation, rapid, and then reduced rate of increase in *R*_h_. An asymptote to a stable radius is not attained even after 2000 s for all three NaCl concentrations.

Like the ULDH-NaCl system, the LDHU-NaCl data display a similar family of curves with an increasing NaCl concentration (see Figure 5c). Time lags for increases in *R*_h_ are slightly compressed relative to the ULDH-NaCl system with the initiation of increased *R*_h_ at 600, 200, and 0 s for 1, 10, and 1000 mM NaCl concentrations, respectively. Moreover, a decline in the increase in *R*_h_ occurs after ca. 100, 600, and 300 s, respectively, for the increasing NaCl concentrations. Thus, as for the ULDH-NaCl system, at 1 and 10 mM NaCl concentrations, the LDHU-NaCl system defines three distinct domains of initiation, rapid, and then reduced rate of increase in *R*_h_, and stable radius was not observed with the samples studied.

The increases in the *R*_h_ of the reference LDH-NaCl and the ULDH-NaCl and LDHU-NaCl systems are broadly consistent with DLVO theory,^49−51^ where an increasing electrolyte concentration leads to the weakening of electrical double-layer repulsion and subsequent nanoparticle aggregation due to predominating van der Waals attraction. Similar colloid behavior, where distinct domains in *R*_h_ develop over time, was previously identified during the aggregation of delaminated LDHs,^52^ which restacked to recover a conventional lamellar LDH nanostructure, with nanoparticle thickness increasing linearly with time with colloid aggregation occurring thereafter.

**Figure 6:**
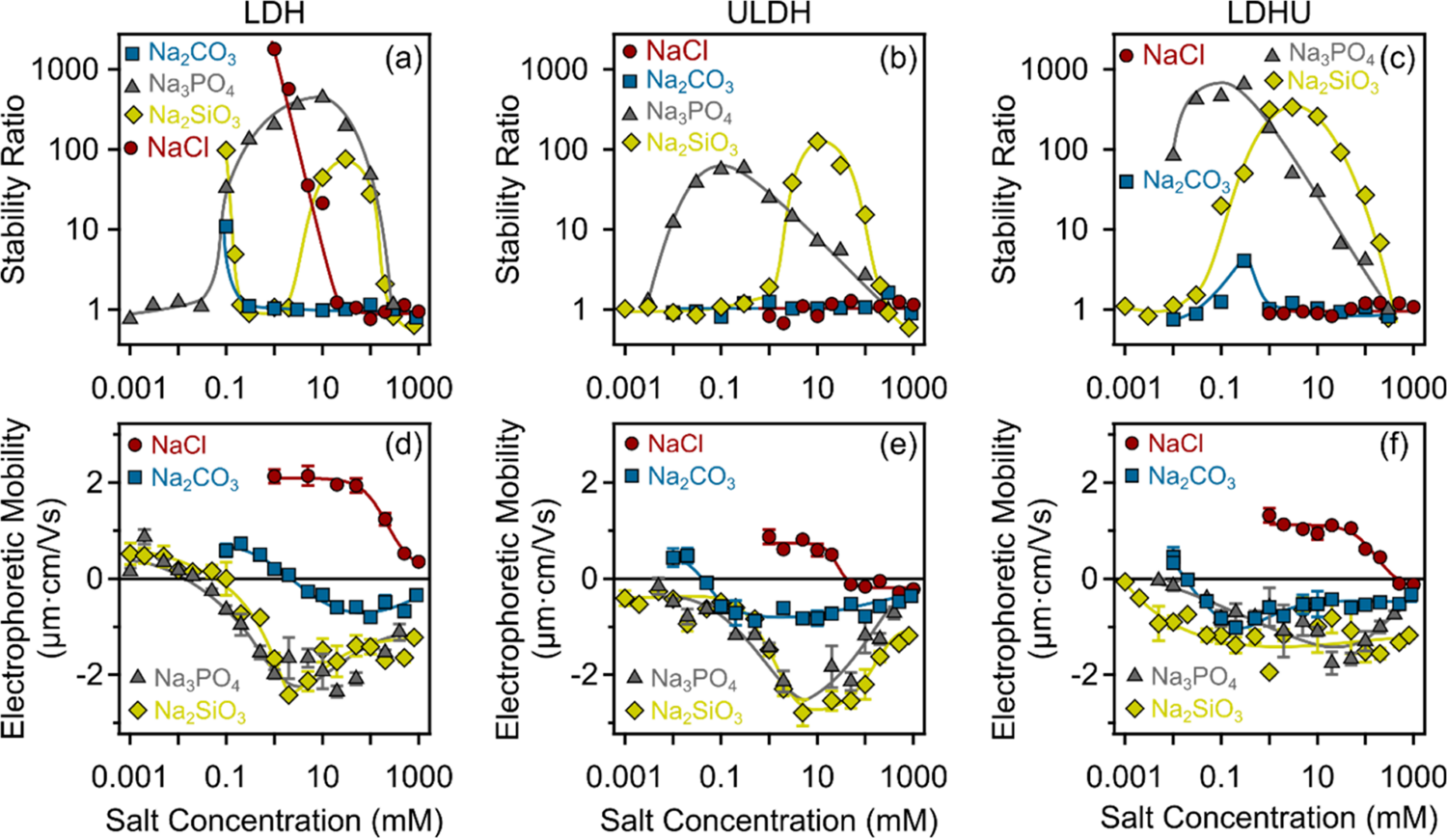
Electrolyte concentration-dependent stability ratios (a–c) and electrophoretic mobilities (d–f) of (a, d) LDH, (b, e) ULDH, and (c, f) LDHU in the presence of NaCl, Na_2_CO_3_, Na_3_PO_4_, and Na_2_SiO_3_. A constant (10 mg/L) nanoparticle concentration was used in all of the experiments. The solid lines are eye guides.

The surface charge measured as electrophoretic mobility (EM) and aggregation features of nanoparticles in LDH, ULDH, and LDHU samples were also compared in the presence of a suite of monovalent and multivalent anion systems, such as NaCl, Na_2_CO_3_, Na_2_SiO_3_, and Na_3_PO_4_ (Figure 6). To quantify the colloidal stability of the dispersions, stability ratios^35,47^ were calculated (Figure 6a–6c) from the *R*_h_ versus time plots (see Figure 5 for some measurements in NaCl solutions). To calculate the stability ratio, the slope determined in 1 M NaCl was divided by the slope obtained in the measurement in question.^53^ Stability ratios of unity correspond to unstable, rapidly aggregating dispersions, while those greater than unity denote more stable environments with slower nanoparticle aggregation.^54^ In the Na_2_CO_3_, Na_2_SiO_3_, and Na_3_PO_4_ systems, when considering stability ratios and EM for the ULDH and LDHU systems in particular, a potential also exists for resolubilization of U as anionic complexes (e.g., UO_2_(CO_3_)_3_^4–^),^55^ or as surface uranyl complexes (e.g., UO_2_(H_2_PO_4_)_2_·3H_2_O),^56^ which may exert fundamental changes to surface charge and aggregation characteristics.

Stability ratios varied fundamentally as a function of the type and concentration of coexisting electrolytes. For the LDH-NaCl system, stability ratios exceeded 1000 at 1 mM but rapidly declined to up to 20 mM and remained at unity thereafter as unstable aggregates. This pattern was broadly similar to that defined by EM, with an initially positive stable charge eventually declining with increasing NaCl concentrations up to 1000 mM (Figure 6d).

The LDH-Na_2_CO_3_ system is closest in behavior to the LDH-NaCl system in that it has an initially unstable region, albeit at a much lower electrolyte concentration of 0.1 mM, and then remains close to unity at all higher electrolyte concentrations. This fundamental change in the stability of these colloidal systems also corresponds to a change from marginally positive to negative EM values of the colloidal nanoparticles.

In contrast to LDH-NaCl, the LDH-Na_3_PO_4_ system maintained the stability ratio near unity up to electrolyte concentrations of 0.1 mM and then described a convex increase in stability ratios of about 500 at 10 mM, decreasing thereafter to unity above about 500 mM. The initial stability ratio close to unity at low electrolyte concentrations is mirrored by a weakly positive EM value, which then became increasingly negative before settling in a concave shape broadly analogous to that of the stability ratio.

The LDH-Na_2_SiO_3_ system is similarly complex to the LDH-Na_3_PO_4_ system with two regions of stability with ratios of ca. 100 at electrolyte concentrations of ca. 0.1 mM and between 10 and 100 mM interspersed by regions of stability ratios of unity corresponding to unstable, rapidly aggregating dispersions. This complex aggregation behavior does not correspond to the surface charge or EM, which displays a similar shape and charge to that of the LDH-Na_3_PO_4_ system.

Concerning the sudden transition between slow and fast aggregation in the LDH-NaCl systems by increasing the salt concentration discussed above, these regimes are separated by the critical coagulation concentration (CCC). This is in line with the findings of previous reports on salt-induced aggregation of LDH particles^29,57,58^ and with the prediction of DLVO theory.^35,49^ Accordingly, at low concentrations, the stability ratios were initially high but exhibited a rapid decrease as the concentration increased (i.e., slow aggregation regime), while at higher concentrations, the stability ratio values of one indicated rapid aggregation (i.e., fast aggregation regime). In the case of ULDH (see Figure 6b) and LDHU (see Figure 6c), no slow aggregation regime could be observed in the presence of NaCl, suggesting that the concentration regime studied exceeded the CCC for the given nanoparticles. Likewise, when Na_2_CO_3_ was present, no CCC could be determined, but there was a slight restabilization, i.e., an increase in the stability ratio data, observed for LDHU. However, in the presence of Na_2_SiO_3_ and Na_3_PO_4_, more complex behavior is identified, with significant restabilization occurring at intermediate concentrations for all nanoparticle types, which can be explained by the tendencies in the charging features as follows.

The EM values were determined under the same conditions as stability ratios (see Figure 6d–f) to understand the colloidal stability regimes. For the NaCl system, the shape of the concentration-dependent mobility curves was similar for all three classes of nanoparticles. With increasing salt concentration, the EM decreased being close to zero at high electrolyte concentrations due to the screening effect on the surface charge.^51^ Notably, the magnitude of the mobilities was markedly higher for LDH (Figure 6d) nanoparticles compared to ULDH (Figure 6e) and LDHU (Figure 6f). This finding supports the high stability observed at low electrolyte levels for LDH (Figure 6a), which can be attributed to sufficiently strong repulsive double-layer forces.

However, the surface charge properties of the nanoparticles are significantly different in the presence of Na_2_CO_3_, Na_2_SiO_3_, and Na_3_PO_4_. At the investigated pH conditions, mostly HCO_3_^–^ and HPO_4_^2–^ ions were present in the system,^59^ while Na_2_SiO_3_ may be present as monomers or from a range of oligomers.

The adsorption of anions in Na_2_CO_3_, Na_2_SiO_3_, and Na_3_PO_4_ initially led to charge neutralization and subsequently charge reversal at higher concentrations, likely due to the strong electrostatic and hydrogen bonding interaction between the anions and LDH nanoparticles.^57,60^ Noteworthy was that the LDH underwent charge neutralization and then reversal at higher concentrations of Na_2_CO_3_, Na_2_SiO_3_, and Na_3_PO_4_. This may reflect that LDH nanoparticles have the highest surface charge compared to the ULDH and LDHU nanoparticles. Finally, the mobilities started to decrease in magnitude at higher electrolyte levels due to the screening effect of the Na^+^ ions on the negative surface charge. This behavior is typical for LDH colloids in electrolyte media.^57,60^

The findings indicate that the aggregation behavior can be explained quantitatively based on the charging features. When Na_2_SiO_3_ or Na_3_PO_4_ is present, there is significant charge reversal, resulting in strong repulsive electric double-layer forces that lead to nanoparticle restabilization. Conversely, in the presence of Na_2_CO_3_, the surface charge after charge inversion is too low to induce consistent restabilization through double-layer repulsion.

These results of the electrophoretic mobility (EM) and aggregation features of LDH, ULDH, and LDHU in the presence of NaCl, Na_2_CO_3_, Na_2_SiO_3_, and Na_3_PO_4_ confirm that UO_2_^2+^ cations in the ULDH and LDHU systems impart similar, fundamental changes in charge and surface chemical features relative to the reference LDH. The implication is that irrespective of whether the UO_2_^2+^ cations are added prior to or following LDH formation, not only is schoepite formed as a separate phase but there is also a similar inhibitory effect on particle growth and surface charge and aggregation characteristics.

### Implications for Nuclear Wastewater Management

This study highlights the complex behavior of LDH colloidal stability as a function of fluid composition and in the presence of elevated UO_2_^2+^ concentrations. This information is directly relevant to the systems where Mg–Al LDH phases are present in U-contaminated natural and engineered systems, including legacy storage facilities at Sellafield, U.K. For example, Sellafield legacy spent nuclear fuel ponds (SNFPs) have highly heterogeneous inventories, which include SNF, cementitious materials, microbes, pond furniture, and extraneous debris. In these systems, Mg–Al LDH phases have the potential to form via the corrosion of Mg-rich fuel cladding, including their potential to form a colloidal phase within these uranium-containing systems.^29^ This study clearly illustrates the effect of changing chemical conditions on the colloidal stability of LDH and ULDH particles and can be used to help predict the extent and duration of colloidal formation in a wide range of systems. This in turn can assist in the development of decommissioning strategies for these types of facilities for multielement/radionuclide removal using in situ-formed LDH-based materials.

## Conclusions

By comparison of the synthesized ULDH systems (ULDH and LDHU) with a control LDH sample (LDH), several important findings were obtained. The incorporation of UO_2_^2+^ resulted in the formation of a new coexisting phase, schoepite, in addition to the typical LDH formation. It was also found that the presence of UO_2_^2+^ cations had a remarkable effect on crystal growth, manifested by a decrease in the LDH size, suggesting a limiting effect on the crystal growth process. Moreover, the aggregation behavior of the synthesized systems was investigated in the presence of different electrolytes (NaCl, Na_2_CO_3_, Na_2_SiO_3_, and Na_3_PO_4_). The results showed a correlation between the aggregation tendency and the charging properties of the colloids. Importantly, this study found that although the UO_2_^2+^ cations did not appear to become an integral part of the LDH structure, they significantly altered the surface properties and thus influenced the aggregation mechanism and dissolution dynamics. These results highlight the complex role of the UO_2_^2+^ cation in LDH systems as a key factor in their behavior under different environmental conditions.

## Supporting Information Available

SAXS, SWAXS, Raman and DLS data (PDF)
