# Supplementary material for: Interaction between Uranyl Cations and Layered Double Hydroxide Nanoparticles: Implications for Nuclear Wastewater Management
Source: ACS ES T Water. 2024 Jun 11;4(7):3059–67. doi: 10.1021/acsestwater.4c00313 (PMC11656702; doi:10.1021/acsestwater.4c00313)
Supplement: Supplementary file 1 — ew4c00313_si_001.pdf [file ew4c00313_si_001.pdf]

Supporting Information (SI)

Interaction between Uranyl Cations and Layered Double Hydroxide Nanoparticles: Implications for Nuclear Wastewater Management

Tamás Péter,[ǂ,ǁ] *Dóra Takács,*[ǂ,ǁ] *Szilárd Sáringer,*[ǂ,ǁ] *Adél Szerlauth,*[ǂ,ǁ] *Kadosa Sajdik,*[ǁ] *Gábor Galbács,*[†] *Matija Tomšič,*[§] *Samuel Shaw,*[⸸] *Katherine Morris,*[⸸] *Grant Douglas,*[*,‡,◊] *and István Szilágyi*[*,ǂ,ǁ]

[ǂ]MTA-SZTE Lendület Biocolloids Research Group, Interdisciplinary Excellence Centre, University of Szeged, H-6720 Szeged, Hungary

[ǁ]Department of Physical Chemistry and Materials Science, University of Szeged, H-6720 Szeged, Hungary

[†]Department of Molecular and Analytical Chemistry, University of Szeged, H-6720 Szeged, Hungary

[§]Faculty of Chemistry and Chemical Technology, University of Ljubljana, Večna pot 113, SI-1000 Ljubljana, Slovenia

[⸸]Research Centre for Radwaste Disposal and Williamson Research Centre, Department of Earth and Environmental Sciences, University of Manchester, UK-M139PL Manchester, United Kingdom

[‡]Centre for Environment and Life Sciences, CSIRO Environment, WA-6913 Wembley, Australia

[◊]School of Molecular and Life Sciences, Curtin University, WA-6102 Bentley, Australia

Details of SAXS and SWAXS Data Analysis. In Figure S1a the experimental SAXS curves of the LDH, ULDH and LDHU samples are shown in a double logarithmic scale representation. The dashed lines represent the curves exhibiting the q^{-3} and q^{-1} slopes. As these scattering curves are still experimentally smeared due to the finite dimensions of the long line-collimated primary X-ray beam, these slopes are analogous to the slopes q^{-4} and q^{-2} on the desmeared SAXS curve, the former characteristic for the so-called Porod regime of the SAXS curve in case of the scattering particles with well-defined spatial boundaries and the latter corresponding to the intermediate regime in the case of the large flat particles (slope q^{0} would correspond to the so-called Guinier regime in the case of spherical particles and the slope q^{-1} in case of cylindrical particles).[1] Indeed, the innermost part of the smeared SAXS curves in Figure S1a appears to have a slope only slightly lower than q^{-1} (analogous to q^{-2} in the desmeared curve, which can be observed in the innermost part of Figure S1b), indicating the presence of the scattering nanostructures with the characteristics of flat particles – large in two dimensions and having a constant thickness. To complement these results, we also numerically desmeared our SAXS curves using the Lake algorithm method[2] and show the obtained desmeared SAXS results in Figure S1b.

To facilitate the non-expert reader in the field of SAXS, we briefly explain the smearing effect, which is characteristic for the SAXS data obtained with the line-collimated SAXS instruments, i.e. the SAXS instruments with X-ray tubes typical for smaller in-house SAXS laboratories. The reason for line collimation of such systems is to significantly improve the statistics of the scattering data, but this is at the expense of the SAXS data, which are correspondingly experimentally smeared. The smearing of the data is the consequence of the finite dimensions of the primary beam, which should ideally be a point. For example, in part arising from the finite length of the primary beam, it is technically described by the following mathematical expression:[3]

$$\widetilde{I}(m) = \int_{-\infty}^{\infty} P(t) \cdot I\left(\sqrt{m^2 + t^2}\right) dt \qquad \text{(S1)}$$

where $\widetilde{I}(m)$ is experimentally smeared scattering function, $I\left(\sqrt{m^2+t^2}\right)$ theoretical (non-smeared) scattering function, $P(t)$ the length profile of primary beam (usually measured experimentally) and t the direction perpendicular to scattering vector q and represents the distance from the center of the primary beam. The desmearing operation on the smeared experimental SAXS data would be an inverse of this integral expression, which is in practice hindered by the infinite integration limits in eq. S1. In practice the problem of desmearing SAXS data can be addressed by the Lake algorithm method,[2] which is iterative by its nature and usually successfully solves the problem of desmearing numerically.

The experimental 2D SWAXS data of the LDH, ULDH, and LDHU samples are shown in Figure S2 and clearly indicate that these powder samples are optically isotropic, as no high-intensity local spots are seen in these images. Slightly downward curved, brighter horizontal regions correspond to the increased scattering intensity of the scattering peaks seen in the XRD spectra and scattering curves in Figures 1a and 1b.

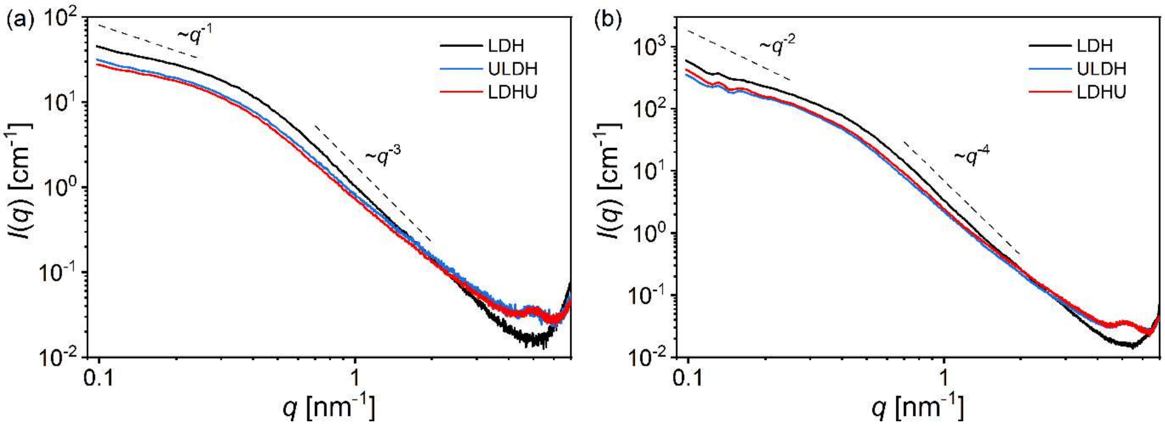

Figure S1. (a) The experimental and (b) desmeared SAXS curve on an absolute scale of LDH, ULDH and LDHU samples in a double logarithmic scale representation.

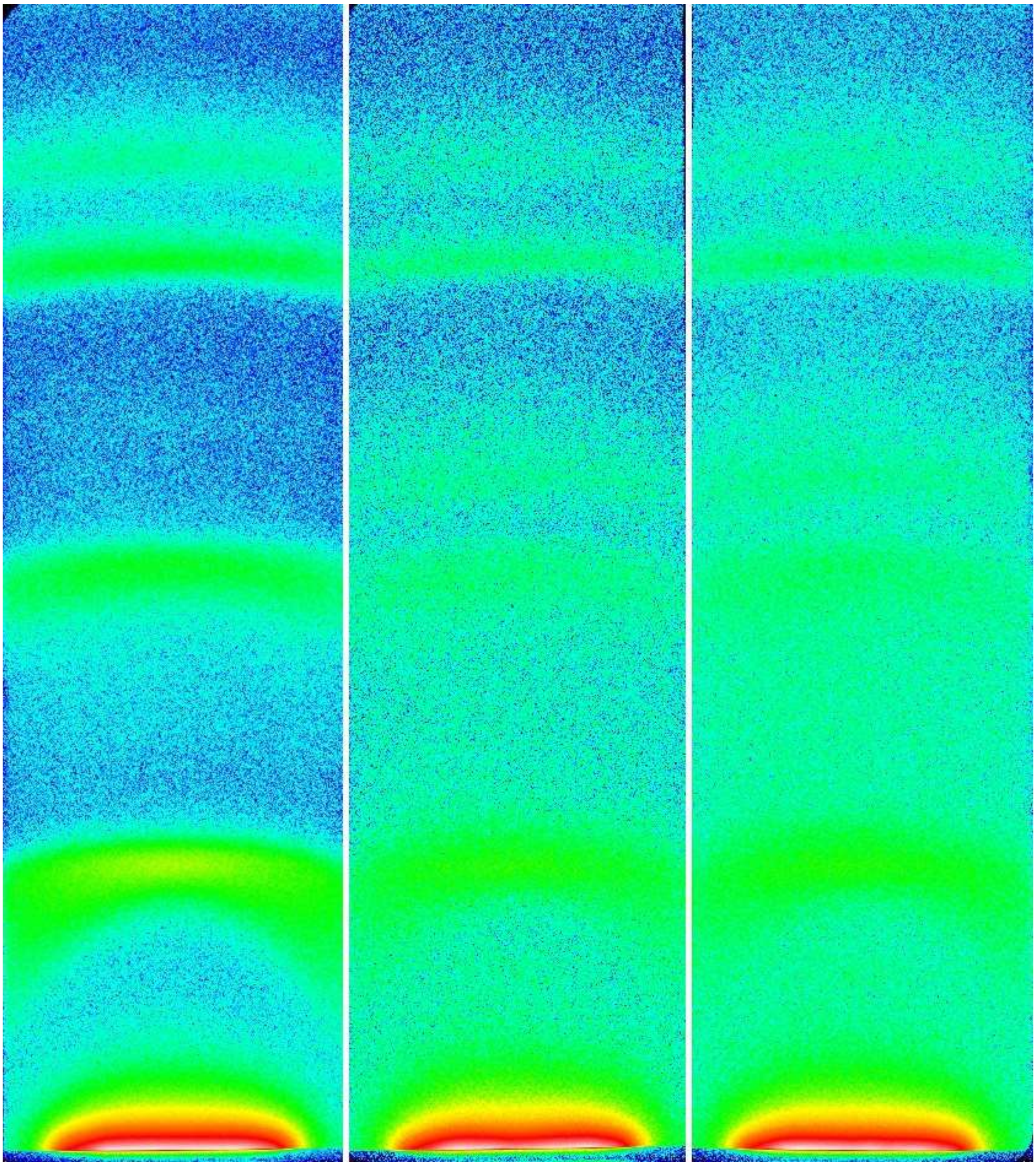

Figure S2. Experimental 2D SWAXS data of (left) LDH, (middle) ULDH, and (right) LDHU samples covering the range of the scattering vector from zero value (lower red part of the images) to about 30.5 nm^{-1} at the top of the images. The red colour represents high intensity, and the dark blue colour represents the lowest intensity.

Table S1. Theoretically (amount of added metal salts) and experimentally (ICP-MS) obtained molar ratios of aluminium, magnesium, and uranium in the samples.

Samples	Theoretical Molar Ratio			ICP-MS Molar Ratio		
	U	Mg	Al	U	Mg	Al
LDH	N/A	3.0	1.0	N/A	3.1	1.0
ULDH	0.1	2.9	1.0	0.1	3.0	1.0
LDHU	0.1	2.9	1.0	0.1	2.9	1.0

Table S2. Assignment of the Raman peaks in the spectra (see Figure 1c) of LDH, ULDH and LDHU materials.

Raman shift (cm^{-1})	Assignment	Sample
1045	ν (NO_3^-)	LDH, LDHU, ULDH
820	ν (UO_2^{2+})	LDHU, ULDH
550	ν (M-O-M)	LDH, LDHU, ULDH

References

1. Glatter, O., *Scattering Methods and their Application in Colloid and Interface Science*. Elsevier: Amsterdam, 2018; p 392.
2. Lake, J. A., An Iterative Method of Slit-Correcting Small Angle X-Ray Data. *Acta Crystallog.* **1967,** *23*, 191-194.
3. Glatter, O., Data treatment. In *Small angle x-ray scattering*, Glatter, O.; Kratky, O., Eds. Academic Press Inc. London Ltd.: London, 1983; pp 119-165.
